# Supplementary material for: Variation in a range of mTOR-related genes associates with intracranial volume and intellectual disability
Source: Nat Commun. 2017 Oct 20;8:1052. doi: 10.1038/s41467-017-00933-6 (PMC5648772; doi:10.1038/s41467-017-00933-6)
Supplement: Supplementary file 1 — Supplementary Information [file 41467_2017_933_MOESM1_ESM.pdf]

SI GUIDE

File Name: Supplementary Information
Description: Supplementary Figures, Supplementary Tables, Supplementary Notes, Supplementary Methods and Supplementary References.

File Name: Supplementary Data 1
Description: All identified de novo mutations

File Name: Supplementary Data 2
Description: Selected MTOR pathway genes

File Name: Supplementary Data 3
Description: Identified de novo mutations in MTOR-related genes

File Name: Supplementary Data 4
Description: OFC per individual

File Name: Supplementary Data 5
Description: Clustering analysis: de novo missense variants

File Name: Supplementary Movie 1
Description: : Movie of a pup IUE at E14.5 with RHEB.P37L, showing a P20 pup with generalized seizure. The white mouse is the dam.

File Name: Peer Review File
Description:

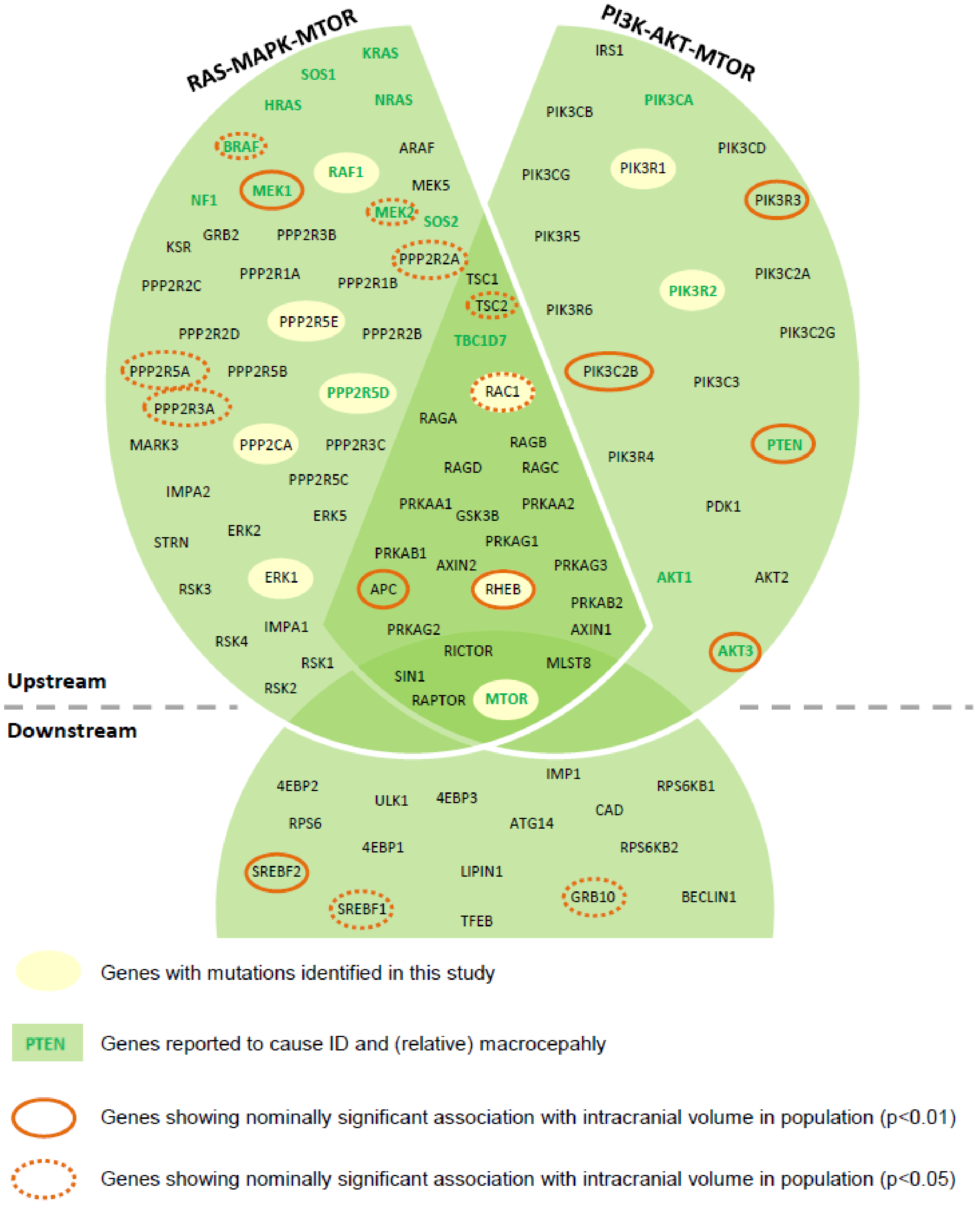

Supplementary Figure 1 Schematic overview of MTOR-related genes showing nominally significant association with intracranial volume in population. Genes (n=101) were included in our MTOR-related gene-set based on three different authorative publications. Both proteins acting upstream of MTOR and proteins acting downstream of MTOR were included. Additionally, we subdivided the total set in two subsets: the RAS-MAPK-MTOR subset and the PI3K-AKT-MTOR subset. In both subsets, downstream genes are included as well. Genes in which we identified *de novo* mutation in this study are marked with a yellow ellipse and genes previously reported to cause ID and (relative) macrocephaly are shown in bolt and dark green. Genes with nominally significant association with ICV in population (n=18) are marked with a bold, orange eclipse ($p<0.01$) or striped, orange eclipse ($p<0.05$).

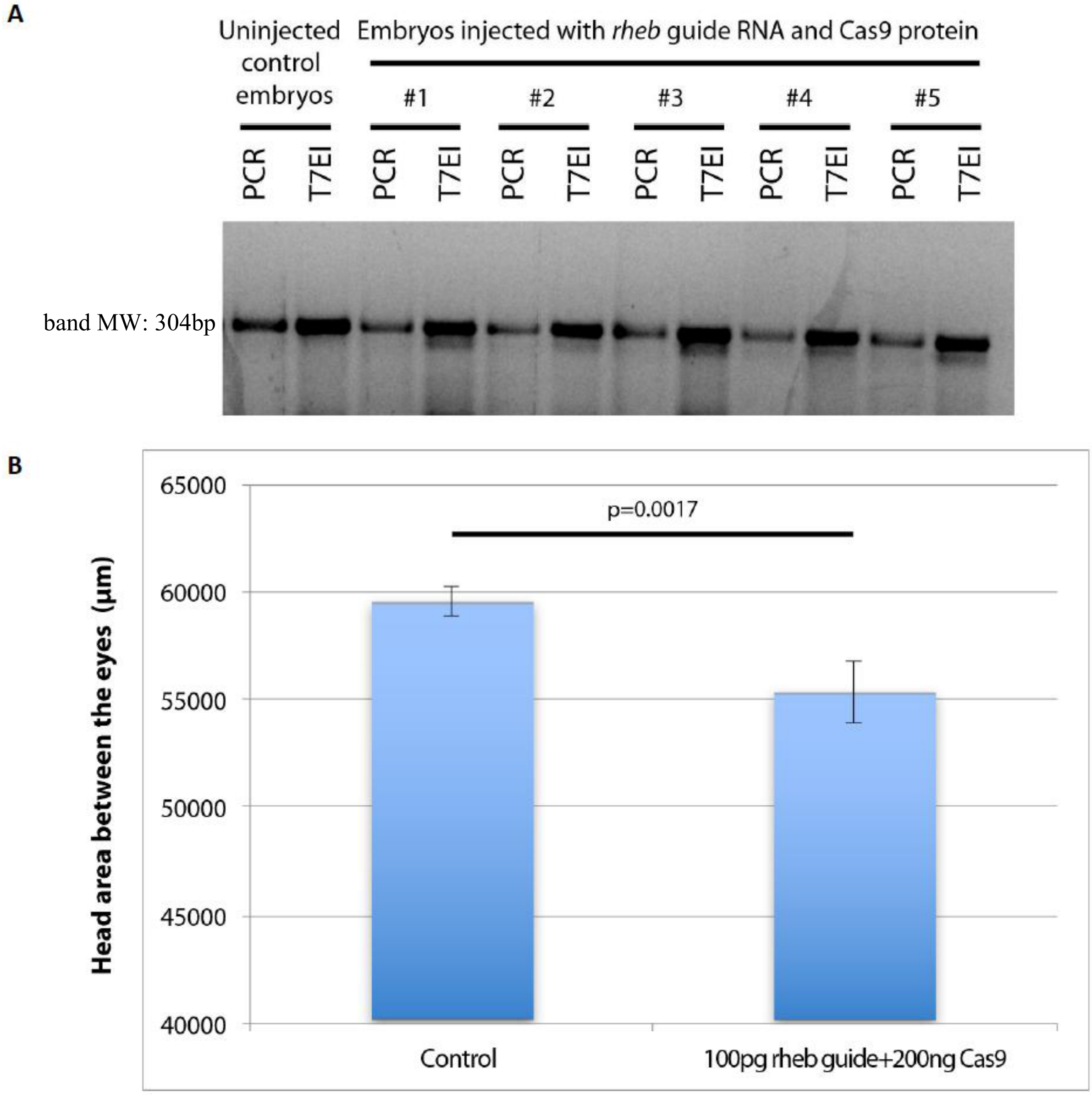

Supplementary Figure 2 Evidence for the efficiency of the CRISPR reagent used to suppress the endogenous expression of *rheb* in developing zebrafish embryos. (**A**) Gel image showing the efficiency of the *rheb* guide RNA following T7 endonuclease assay evaluation (band MW: 304bp). The first two lanes show control amplicons from the locus flanking the targeted sequence, with no aberrations observed. In the embryos injected with *rheb* guide RNA and Cas9 protein, aberrations are evident for embryos #1 - #5, showing that the guide is efficiently introducing sequence aberrations in all injected embryos. PCR fragments from 4 *rheb* embyos with a positive T7 assay were cloned into the pCR4/TOPO TA cloning vector (Life technologies), and 40 clonies from each cloned embryo were Sanger sequenced. Sequence aberrations were observed in ~75% of the evaluated *rheb* clones. (**B**) Bar graph showing the quantification of the headsize phenotype in control embryos and larvae injected with *rheb* guide RNA and Cas9. Statistical analyses were performed by Student`s t-test.

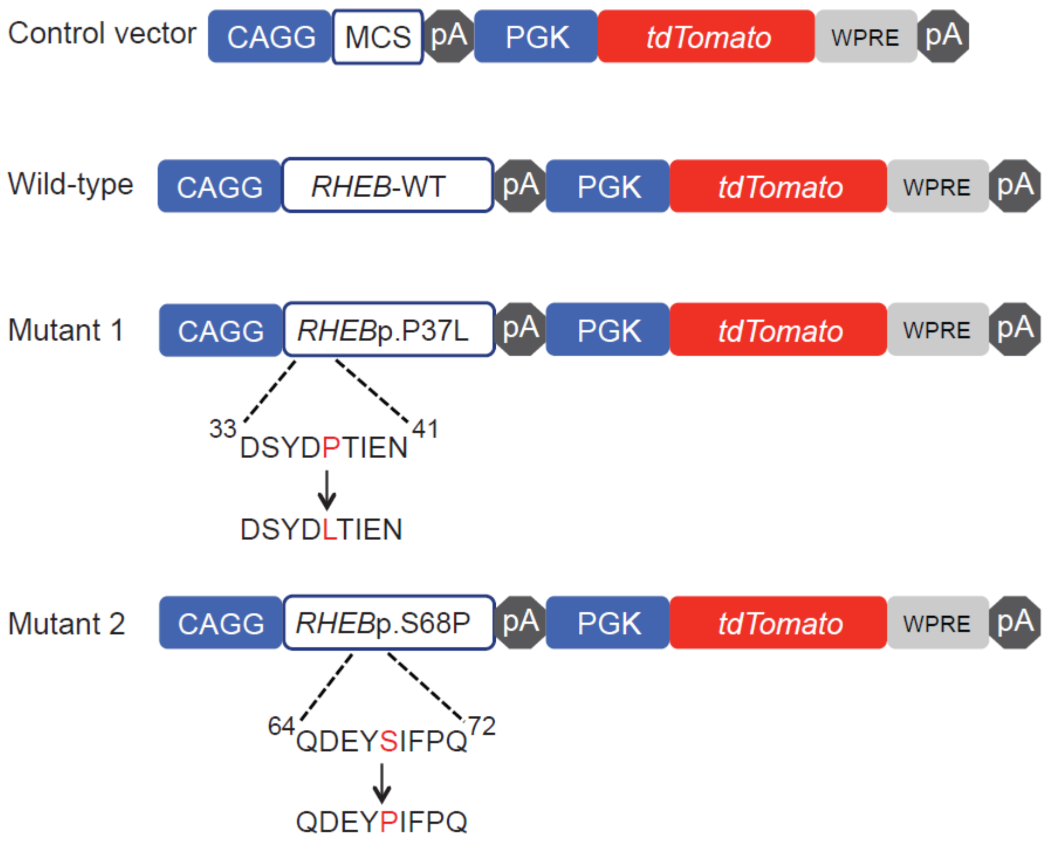

Supplementary Figure 3 Schematic representation of the dual-promotor expression vector with the different RHEB constructs. Schematic representation of the control vector consisting of a *CAGG* promoter followed by a multiple cloning site (MCS) to insert genes of interest. The *tdTomato* gene is expressed under the *PGK* promoter, to allow for independent expression of both the gene of interest and *tdTomato*.

SUPPLEMENTARY TABLES

Supplementary Table 1: Statistical enrichment analysis of de novo mutations in MTOR-related genes

Gene name	Expected gene-specific mutation rate	Number functional DNMs	Functional p-value	Bonferroni corrected functional p-value
AKT1	2.21E-05	0	1	1
AKT2	2.09E-05	0	1	1
AKT3	1.46E-05	0	1	1
APC	8.18E-05	0	1	1
ARAF	2.51E-05	0	1	1
ATG14	1.91E-05	0	1	1
AXIN1	3.47E-05	0	1	1
AXIN2	3.35E-05	0	1	1
BECN1	1.52E-05	0	1	1
BRAF	2.31E-05	0	1	1
CAD	8.79E-05	0	1	1
EIF4EBP1	2.83E-06	0	1	1
EIF4EBP2	5.17E-06	0	1	1
EIF4EBP3	2.39E-06	0	1	1
GRB10	1.89E-05	0	1	1
GRB2	8.44E-06	0	1	1
GSK3B	1.48E-05	0	1	1
HRAS	7.87E-06	0	1	1
IMMP1L	5.23E-06	0	1	1
IMPA1	8.26E-06	0	1	1
IMPA2	1.12E-05	0	1	1
KRAS	5.20E-06	0	1	1
KSR1	3.09E-05	0	1	1
LPIN1	3.12E-05	0	1	1
MAP2K1	1.20E-05	0	1	1
MAP2K2	1.43E-05	0	1	1
MAP2K5	1.32E-05	0	1	1
MAPK1	9.81E-06	0	1	1
MAPK3	1.48E-05	1	0.024153414	1
MAPK7	3.01E-05	0	1	1
MAPKAP1	1.84E-05	0	1	1
MARK3	2.65E-05	0	1	1
MLST8	1.54E-05	0	1	1
MTOR	9.24E-05	1	0.141527932	1
NF1	8.54E-05	0	1	1
NRAS	5.78E-06	0	1	1
PDK1	1.40E-05	0	1	1

PIK3C2A	4.81E-05	0	1	1
PIK3C2B	6.00E-05	0	1	1
PIK3C2G	3.75E-05	0	1	1
PIK3C3	2.77E-05	0	1	1
PIK3CA	3.27E-05	0	1	1
PIK3CB	3.27E-05	0	1	1
PIK3CD	4.42E-05	0	1	1
PIK3CG	4.05E-05	0	1	1
PIK3R1	2.39E-05	2	0.000758647	0.076623383
PIK3R2	2.48E-05	1	0.040082839	1
PIK3R3	1.53E-05	0	1	1
PIK3R4	4.25E-05	0	1	1
PIK3R5	2.07E-05	0	1	1
PIK3R6	0.001017849	0	1	1
PPP2CA	1.12E-05	2	0.000167888	0.016956732
PPP2R1A	2.18E-05	0	1	1
PPP2R1B	2.03E-05	0	1	1
PPP2R2A	1.31E-05	0	1	1
PPP2R2B	1.65E-05	0	1	1
PPP2R2C	1.92E-05	0	1	1
PPP2R2D	1.58E-05	0	1	1
PPP2R3A	3.17E-05	0	1	1
PPP2R3B	2.22E-05	0	1	1
PPP2R3C	1.30E-05	0	1	1
PPP2R5A	1.49E-05	0	1	1
PPP2R5B	1.73E-05	0	1	1
PPP2R5C	1.67E-05	0	1	1
PPP2R5D	2.20E-05	3	7.75E-06	0.000783174
PPP2R5E	1.47E-05	1	0.023980694	1
PRKAA1	1.76E-05	0	1	1
PRKAA2	1.71E-05	0	1	1
PRKAB1	1.08E-05	0	1	1
PRKAB2	7.86E-06	0	1	1
PRKAG1	9.64E-06	0	1	1
PRKAG2	2.18E-05	0	1	1
PRKAG3	1.72E-05	0	1	1
PTEN	1.20E-05	0	1	1
RAC1	7.48E-06	2	7.58E-05	0.007657126
RAF1	2.27E-05	1	0.036735716	1
RHEB	5.74E-06	2	4.47E-05	0.004514218
RICTOR	5.23E-05	0	1	1

RPS6	1.03E-05	0	1	1
RPS6KA1	2.70E-05	0	1	1
RPS6KA2	3.07E-05	0	1	1
RPS6KA3	2.22E-05	0	1	1
RPS6KA6	2.23E-05	0	1	1
RPS6KB1	1.72E-05	0	1	1
RPS6KB2	2.08E-05	0	1	1
RPTOR	5.93E-05	0	1	1
RRAGA	1.36E-05	0	1	1
RRAGB	1.32E-05	0	1	1
RRAGC	9.12E-06	0	1	1
RRAGD	1.03E-05	0	1	1
SOS1	4.05E-05	0	1	1
SOS2	4.08E-05	0	1	1
SREBF1	4.06E-05	0	1	1
SREBF2	4.03E-05	0	1	1
STRN	2.22E-05	0	1	1
TBC1D7	1.05E-05	0	1	1
TFEB	1.70E-05	0	1	1
TSC1	3.88E-05	0	1	1
TSC2	7.36E-05	0	1	1
ULK1	4.89E-05	0	1	1
IRS1	5.31E-05	0	1	1
**mTOR pathway**	**3.51E-03**	**16**	**0.000350082**	

Supplementary Table 2: P-values of MTOR-related genes with recurrent *de novo* missense mutations

Gene name	Transcript	Coding size	Mutation location cDNA	P-value clustering analysis
MTOR	NM_004958	7650	4375, 4555, 5395	0.060619394
PPP2R5D	NM_006245	1809	157, 592, 592, 592, 592, 592, 592, 592, 592, 592, 592, 598, 602, 619, 752, 1258	1,00E-01
RHEB	NM_005614	555	110, 202	0.304626954
PIK3R1	NM_181523	2175	1359, 1614, 1692	0.0900091
RAC1	NM_018890	636	53, 116, 151, 190, 218	0.04898951

Supplementary Table 3: Known MTOR-related ID genes and association with aberrant brain size

Gene	Known ID gene?	Known aberrant brain size?	Reference
PIK3CA	Yes	(Hemi) megalencephaly	Riviere J.B., Mirzaa G.M., O'Roak B.J. et al (2012), Nature Genet. 44: 934-940
PIK3R1	Yes	Low-normal; Microcephaly	Thauvin-Robinet C., Auclair M., Duplomb L. et al (2013), Am. J. Hum. Genet. 93: 141-149
PIK3R2	Yes	Megalencephaly	Riviere J.B., Mirzaa G.M., O'Roak B.J. et al (2012), Nature Genet. 44: 934-940
PTEN	Yes	Megalencephaly	Liaw, D., Marsh, D. J., Li, J. et al (1997), Nature Genet. 16: 64-67
AKT1	Yes	Megalencephaly	Orloff M. S., He X., Peterson C. et al (2013), Am. J. Hum. Genet. 92: 76-80
AKT3	Yes	(Hemi) megalencephaly	Riviere J.B., Mirzaa G.M., O'Roak B.J. et al (2012), Nature Genet. 44: 934-940
TSC1	Yes	Normal	van Slegtenhorst M., de Hoogt R., Hermans C. et al (1997), Science 277: 805-808
TSC2	Yes	Normal	Kumar A., Wolpert C., Kandt R. S. et al (1995), Hum. Molec. Genet. 4: 1471-1472
TBC1D7	Yes	Megalencephaly	Capo-Chichi J.-M., Tcherkezian J., Hamdan F. F. (2013), J. Med. Genet. 50: 740-744
MTOR	Yes	Megalencephaly	Smith L. D., Saunders C. J., Dinwiddie D. L et al (2013), J. Genomes Exomes 2: 63-72
SOS1	Yes	Megalencephaly	Roberts A. E., Araki T., Swanson K. D. et al (2007), Nature Genet. 39: 70-74
SOS2	Yes	Relative macocephaly	Yamamoto G. L., Aguena M., Gos M. et al (2015), J. Med. Genet. 52: 413-421
KRAS	Yes	Relative macocephaly	Schubbert S., Zenker M., Rowe S. L. et al (2006), Nature Genet. 38: 331-336
HRAS	Yes	Megalencephaly	Aoki Y., Niihori T., Kawame H. et al (2005), Nature Genet. 37: 1038-1040
NRAS	Yes	Megalencephaly	Cirstea I. C., Kutsche K., Dvorsky R. et al (2010), Nature. Genet. 42: 27-29
B-RAF	Yes	Relative macocephaly	Sarkozy A., Carta C., Moretti S. et al (2009), Hum. Mutat. 30: 695-702
RAF-1	Yes	Relative macocephaly	Pandit B., Sarkozy A., Pennacchio L. A et al (2007), Nature Genet. 39: 1007-1012
NF1	Yes	Megalencephaly	Upadhyaya M., Shen M., Cherryson A. et al (1992), Hum. Molec. Genet. 1: 735-740
MEK-1 (MAP2K1/MKK1)	Yes	Relative macocephaly	Rodriguez-Viciana P., Tetsu O., Tidyman W. E. et al (2006), Science 311: 1287-1290
MEK-2 (MAP2K2/MKK2)	Yes	Relative macocephaly	Rodriguez-Viciana P., Tetsu O., Tidyman W. E. et al (2006), Science 311: 1287-1290
PPP2R1A (PR65a)	Yes	Microcephaly	Houge G., Haesen D., Vissers L. E. L. M. et al (2015), J. Clin. Invest. 125: 3051-3062
PPP2R5D (PR61D)	Yes	Megalencephaly; Microcephaly	Houge G., Haesen D., Vissers L. E. L. M. et al (2015), J. Clin. Invest. 125: 3051-3062
RSK-2 (RPS6KA3)	Yes	Microcephaly	Trivier E., De Cesare D., Jacquot S. et al (1996), Nature 384: 567-570

Supplementary Table 4: Subdivision of genes in two major branches of the MTOR pathway

RAS-MAPK-MTOR branch	PI3K-AKT-MTOR branch
3845	3667
3265	5290
4893	5291
369	5293
673	5294
5894	5295
4763	5296
5604	8503
5605	23533
5607	146850
8844	5286
5518	5287
5519	5288
5520	5289
5521	30849
5522	5728
55844	5163
5525	207
5526	208
5527	10000
5528	7248
5529	7249
5523	51256
28227	6009
55012	64223
6801	2475
5515	57521
4140	10670
3612	10325
3613	64121
5595	58528
5594	324
5598	8312
6195	8313
6197	2932
6196	5562
27330	5564
7248	5571
7249	5563
51256	5565
6009	51422
64223	53632
2475	5879
57521	79109
10670	253260
10325	8408
64121	6198
58528	6199
22863	1978
196294	1979
8312	8637
8313	2887
2932	6194

5562	23175
5564	6720
5571	6721
5563	790
5565	7942
51422	8678
53632	22863
5879	196294
79109	
253260	
8408	
6198	
6199	
1978	
1979	
8637	
2887	
6194	
23175	
6720	
6721	
790	
7942	
8678	
2885	
6654	
6655	
324	

Supplementary Table 5: Gene-based results of Reactome_PI3K_AKT_activation gene-set using the MAGMA software

GENE	Genename	CHR	START	STOP	NSNPS	EFF_SIZE	STAT	P
2309	FOXO3	6	108781026	1,09E+08	746	298	5,9392	1,43E-09
10000	AKT3	1	243551535	2,44E+08	861	320	4,0829	2,22E-05
387	RHOA	3	49296578	49549526	382	159	3,6811	0,000116
2931	GSK3A	19	42634338	42846736	243	87	2,7503	0,002977
5728	PTEN	10	89523195	89828532	681	320	2,7199	0,003265
5295	PIK3R1	5	67411584	67697649	1017	432	2,6298	0,004271
1147	CHUK	10	101848124	1,02E+08	621	219	2,6184	0,004417
3164	NR4A1	12	52316616	52553291	675	342	2,2467	0,012331
117145	THEM4	1	151743342	1,52E+08	620	216	2,1803	0,014617
7249	TSC2	16	1997990	2238713	604	319	1,7776	0,037731
2308	FOXO1	13	41029801	41340734	822	350	1,7677	0,038554
6199	RPS6KB2	11	67095935	67302879	309	139	1,5657	0,058706
1385	CREB1	2	208294616	2,09E+08	683	315	1,4066	0,07977
253260	RICTOR	5	38838022	39174501	878	301	1,4034	0,080253
64223	MLST8	16	2155178	2359418	343	193	1,3807	0,083687
1026	CDKN1A	6	36544237	36755116	893	366	1,3584	0,087167
4193	MDM2	12	69101971	69339212	657	313	1,2487	0,10589
79109	MAPKAP1	9	128099673	1,29E+08	1043	438	1,1962	0,11581
5291	PIK3CB	3	138274231	1,39E+08	468	167	1,0603	0,1445
4914	NTRK1	1	156685542	1,57E+08	606	238	1,045	0,14802
1027	CDKN1B	12	12770302	12975305	515	263	0,87281	0,19138
8660	IRS2	13	110306184	1,11E+08	857	410	0,72505	0,23421
207	AKT1	14	105135686	1,05E+08	676	306	0,70967	0,23895
5170	PDPK1	16	2487970	2753189	235	125	0,17362	0,43108
5296	PIK3R2	19	18164016	18381343	739	295	0,050073	0,48003
57761	TRIB3	20	261308	478203	729	417	0,043647	0,48259
4803	NGF	1	115728537	1,16E+08	977	370	-0,00436	0,50174

842	CASP9	1	15718769	15951285	753	242	-0,02734	0,51091
84335	AKT1S1	19	50272296	50480644	596	279	-0,03236	0,51291
3667	IRS1	2	227496033	2,28E+08	593	219	-0,17806	0,57066
23239	PHLPP1	18	60282672	60747666	1147	514	-0,23892	0,59442
2475	MTOR	1	11066588	11422608	776	172	-0,35988	0,64053
572	BAD	11	63937300	64152176	451	205	-0,42081	0,66305
208	AKT2	19	40636224	40891265	651	255	-0,88001	0,81057
5290	PIK3CA	3	178766311	1,79E+08	832	338	-1,5135	0,93492

Grey gene was also included in the expert-curated mTOR gene-set

Supplementary Table 6: Gene-based results of 96 genes belonging to the MTOR gene-set using the MAGMA software

GENE	GENE_NAME	CHR	START	STOP	NSNPS	EFF_SIZE	STAT	P
10000	AKT3	1	2,44E+08	2,44E+08	861	320	4,0829	2,22E-05
324	APC	5	1,12E+08	1,12E+08	1195	346	3,3416	0,000417
5594	MAPK1	22	22013946	22321970	911	385	3,157	0,000797
6721	SREBF2	22	42129106	42402375	732	291	2,9106	0,001804
8503	PIK3R3	1	46405812	46698708	537	169	2,7336	0,003132
5728	PTEN	10	89523195	89828532	681	320	2,7199	0,003265
6009	RHEB	7	1,51E+08	1,51E+08	770	362	2,6431	0,004107
5295	PIK3R1	5	67411584	67697649	1017	432	2,6298	0,004271
5287	PIK3C2B	1	2,04E+08	2,05E+08	727	277	2,5197	0,005872
673	BRAF	7	1,4E+08	1,41E+08	724	222	2,161	0,015346
2887	GRB10	7	50557760	50961159	1462	504	2,0816	0,018688
5879	RAC1	7	6314126	6543598	956	425	2,0013	0,022682
5523	PPP2R3A	3	1,36E+08	1,36E+08	681	294	1,9849	0,023575
5520	PPP2R2A	8	26049007	26330196	1052	490	1,8433	0,032643
5605	MAP2K2	19	3990319	4224126	642	313	1,7979	0,036094
7249	TSC2	16	1997990	2238713	604	319	1,7776	0,037731
5525	PPP2R5A	1	2,12E+08	2,13E+08	774	307	1,7725	0,038157
6720	SREBF1	17	17614663	17840325	338	162	1,6969	0,044858
23175	LPIN1	2	11786740	12067535	996	508	1,5968	0,05516
6199	RPS6KB2	11	67095935	67302879	309	139	1,5657	0,058706
4140	MARK3	14	1,04E+08	1,04E+08	884	295	1,4962	0,067301
253260	RICTOR	5	38838022	39174501	878	301	1,4034	0,080253
64223	MLST8	16	2155178	2359418	343	193	1,3807	0,083687
8312	AXIN1	16	237440	502676	882	482	1,3013	0,096576
79109	MAPKAP1	9	1,28E+08	1,29E+08	1043	438	1,1962	0,11581
58528	RRAGD	6	89974335	90221995	709	374	1,1949	0,11606
6198	RPS6KB1	17	57870443	58127787	308	121	1,0673	0,14291

5291	PIK3CB	3	1,38E+08	1,39E+08	468	167	1,0603	0,1445
8313	AXIN2	17	63424681	63657740	672	301	0,99861	0,15899
5526	PPP2R5B	11	64592143	64801950	462	199	0,99169	0,16067
51422	PRKAG2	7	1,51E+08	1,52E+08	1829	868	0,94281	0,17289
8408	ULK1	12	1,32E+08	1,33E+08	630	320	0,92147	0,1784
4763	NF1	17	29321945	29804695	698	259	0,92131	0,17844
30849	PIK3R4	3	1,3E+08	1,31E+08	536	167	0,84709	0,19847
57521	RPTOR	17	78418625	79040173	2580	1004	0,83039	0,20316
5518	PPP2R1A	19	52593055	52829678	963	361	0,7996	0,21197
1979	EIF4EBP2	10	72063861	72288374	736	335	0,76398	0,22244
6801	STRN	2	36964841	37293615	976	447	0,76315	0,22269
10670	RRAGA	9	18949372	19151023	906	322	0,74297	0,22875
207	AKT1	14	1,05E+08	1,05E+08	676	306	0,70967	0,23895
8637	EIF4EBP3	5	1,4E+08	1,4E+08	281	127	0,61756	0,26843
5522	PPP2R2C	4	6222305	6665327	1684	782	0,49929	0,30879
790	CAD	2	27340258	27566654	437	181	0,44216	0,32919
6194	RPS6	9	19276254	19480235	535	261	0,35128	0,36269
5163	PDK1	2	1,73E+08	1,74E+08	921	277	0,32056	0,37427
5293	PIK3CD	1	9611790	9889172	455	254	0,29966	0,38222
1978	EIF4EBP1	8	37788020	38017883	264	108	0,28672	0,38716
55012	PPP2R3C	14	35454678	35691519	851	286	0,27276	0,39252
4893	NRAS	1	1,15E+08	1,15E+08	504	225	0,26948	0,39378
5563	PRKAA2	1	57010990	57281008	927	332	0,24592	0,40287
5294	PIK3CG	7	1,06E+08	1,07E+08	732	338	0,24467	0,40336
6654	SOS1	2	39108690	39447604	727	296	0,23446	0,40731
7942	TFEB	6	41551716	41803997	745	379	0,2306	0,40881
3845	KRAS	12	25258180	25503854	818	253	0,20824	0,41752
64121	RRAGC	1	39205005	39425340	654	305	0,20159	0,42012
53632	PRKAG3	2	2,2E+08	2,2E+08	320	95	0,15254	0,43938
5515	PPP2CA	5	1,33E+08	1,34E+08	451	162	0,12783	0,44914

5564	PRKAB1	12	1,2E+08	1,2E+08	512	197	0,077046	0,46929
5286	PIK3C2A	11	17008122	17291354	705	240	0,070221	0,47201
5296	PIK3R2	19	18164016	18381343	739	295	0,050073	0,48003
5528	PPP2R5D	6	42852330	43080080	600	232	0,005518	0,4978
8844	KSR1	17	25699036	26050718	1006	363	0,002564	0,49898
5288	PIK3C2G	12	18314474	18901352	2012	675	-0,04873	0,51943
5595	MAPK3	16	30025426	30234630	236	120	-0,14791	0,55879
3667	IRS1	2	2,27E+08	2,28E+08	593	219	-0,17806	0,57066
5607	MAP2K5	15	67735021	68199455	1177	413	-0,20467	0,58109
7248	TSC1	9	1,36E+08	1,36E+08	915	456	-0,31862	0,62499
2475	MTOR	1	11066588	11422608	776	172	-0,35988	0,64053
55844	PPP2R2D	10	1,34E+08	1,34E+08	604	298	-0,41835	0,66215
3612	IMPA1	8	82469151	82698589	789	265	-0,47181	0,68147
2932	GSK3B	3	1,19E+08	1,2E+08	1055	424	-0,49086	0,68824
196294	IMMP1L	11	31353949	31631169	395	163	-0,49886	0,69106
6195	RPS6KA1	1	26756249	27001520	416	205	-0,54542	0,70727
5289	PIK3C3	18	39435199	39761448	718	240	-0,56914	0,71537
5565	PRKAB2	1	1,47E+08	1,47E+08	1027	263	-0,5724	0,71647
6655	SOS2	14	50483846	50798099	922	349	-0,67363	0,74973
8678	BECN1	17	40862150	41076310	218	130	-0,87324	0,80873
208	AKT2	19	40636224	40891265	651	255	-0,88001	0,81057
5562	PRKAA1	5	40659481	40898297	636	258	-0,92259	0,82189
5598	MAPK7	17	19181034	19386857	318	103	-0,95597	0,83046
5604	MAP2K1	15	66579211	66883882	1016	333	-0,96495	0,83271
3613	IMPA2	18	11881427	12130885	1123	425	-1,0587	0,85513
146850	PIK3R6	17	8606055	8870994	770	351	-1,0908	0,86232
23533	PIK3R5	17	8682233	8969024	773	400	-1,1182	0,86826
6196	RPS6KA2	6	1,67E+08	1,67E+08	2152	898	-1,1346	0,87173
5521	PPP2R2B	5	1,46E+08	1,47E+08	1705	598	-1,2241	0,88954
5527	PPP2R5C	14	1,02E+08	1,02E+08	1045	346	-1,2574	0,89569

5519	PPP2R1B	11	1,11E+08	1,12E+08	431	145	-1,3095	0,90482
22863	ATG14	14	55733109	55978576	958	238	-1,4038	0,91981
5529	PPP2R5E	14	63741355	64110079	945	332	-1,4202	0,92223
2885	GRB2	17	73214157	73501790	679	246	-1,5017	0,93342
5290	PIK3CA	3	1,79E+08	1,79E+08	832	338	-1,5135	0,93492
5571	PRKAG1	12	49296055	49512629	365	147	-1,5211	0,93588
51256	TBC1D7	6	13205183	13428787	777	205	-1,5707	0,94188
5894	RAF1	3	12525100	12805700	844	295	-1,5789	0,94282
3265	HRAS	11	432242	635550	857	448	-1,583	0,94329

Grey gene was also included in the Reactome_PI3K_AKT_activation gene-set

Supplementary Table 7: Clinical information of patients with de novo mutations in *RHEB*

**Clinical details of individuals with *de novo RHEB* mutations**			
	Family 1	*Family 2*	
	**Individual 1**	**Individual 2**	**Individual 3**
Gender	Female	Male	Female
Age of last visit	3.5 years	29 years	27 years
Ethnicity	Caucasian	Caucasian	Caucasian
**Mutations**			
cDNA change	c.202T>C	c.110C>T	c.110C>T
Amino acid change	p.Ser68Pro	p.Pro37Leu	p.Pro37Leu
Chromosome position (Hg 19)	Chr7:151174492	Chr7:151188043G>A	Chr7:151188043G>A
**Growth**			
Height	100.5 cm (-2 SD)	171cm (-2 SD)	151 cm (-3 SD)
Weight (-height)	NR	18 years: 47 kg (-1.5 SD)	17 years: 54 kg (+2 SD)
Head circumference	3.5 years: 53.4 cm (+2.5 SD)	5 years: 56.5 cm (+3 SD)	0.5 years: 50 cm (+3 SD)
		29 years: 60 cm (+1.5 SD)	23 years: 57 cm (+1 SD)
ICD	3,6 cm (+3 SD)	4 cm (+3.5 SD)	3.6 cm (+2 SD)
OCD	8.1 cm (+0.5 SD)	10 cm (+2 SD)	10 cm (+2 SD)
**Development**			
Intellectual disability	Severe-profound ID	Severe-profound ID	Severe ID
**Neurologic**			
Epilepsy	+	-	+
Hypotonia	+	+	+
Polyneuropathy		+	-
Spasticity	-	+	+
**Behavior**			
Autistic features	+	+	+
Sleep disturbances	-	+	-
**Skeletal**			
Scoliosis/kyphosis	-	+	+
Recurrent hipdislocation	-	+	+
**Cardial**			
Cardiac abnormality	-	+	-
**Brain imaging**	*MRI*<br>*Re-evaluation*	*Ultrasound*<br>*Report*	*CT*
Megalencephaly	+	+	NR
Dilatation lateral ventricles	+	+	+
Hypoplastic cerebellum	+	+	NR

Abbreviations: NR= Not Reported; SD = Standard Deviation

Supplementary Table 8: Information on sampling and demographics of study populations

All Subjects								Patients Excluded (Healthy Only)			
**Cohort**	**Analysis**	**Study Design**	**Ancestry**	**Total N**	**N Females**	**Mean Age (SD)**	**Age Range**	**Total N**	**N Females**	**Mean Age (SD)**	**Age Range**
**3C-Dijon (CHARGE)**	CHARGE	Population-based	European	1403	882	72.2 (4.1)	65 - 82	-	-	-	-
**AddNeuroMed**	ENIGMA	Case-control (AD, MCI and health controls)	European	357	204	74.4 (6.4)	53 - 90	114	65	72.8 (6.8)	53 - 88
**ADNI**	ENIGMA	Case-Control (AD, MCI, healthy control)	European	747	302	75.4 (6.9)	55 - 91	204	93	76.1 (5.0)	60 - 89
**ADNI2GO**	ENIGMA	Case-Control (AD, MCI, healthy control)	European	362	203	72.8 (7.4)	55 - 91	337	186	72.6 (7.1)	55 - 91
**AGES (CHARGE)**	CHARGE	Population-based	European	2510	1506	75.95 (5.30)	66 - 95	-	-	-	-
**ARIC**	CHARGE	Population-based	European	413	253	72.71 (4.33)	62 - 82	-	-	-	-
**ASPS (CHARGE)**	CHARGE	Population-based	European	172	120	69.8 (6.7)	52 - 84	-	-	-	-
**ASPSFam (CHARGE)**	CHARGE	Population-based	European	339	205	65.2 (10.5)	38 - 86	-	-	-	-
**Betula**	ENIGMA	Population-based	European	353	185	62.3 (13.3)	25 - 95	-	-	-	-
**BFS**	ENIGMA	Population-based	European	220	115	24 (7.9)	15 - 60	-	-	-	-
**BIG**	ENIGMA	Population-based	European	1300	747	22.9 (3.8)	18 - 41	-	-	-	-
**BrainSCALE**	ENIGMA	Population-based Twin Study	European	277	147	10.0 (1.3)	9 - 15	-	-	-	-
**BRCDECC**	ENIGMA	Case-control (MDD and healthy controls)	European	169	105	49.9 (8.6)	26 - 71	79	44	51.3 (7.7)	26 - 66
**CHS**	CHARGE	Population-	European	648	398	78.89 (4.2)	73 - 95	-	-	-	-

**(CHARGE)**		based									
**EPIGEN**	ENIGMA	Epilepsy cases	European	233	138	38.5 (12.7)	14 - 85	Patients-only			
**ERF (CHARGE)**	CHARGE	Family-based study	European	118	60	64.3 (4.5)	55 - 76	-	-	-	-
**FHS (CHARGE)**	CHARGE	Population-based	European	938	534	58.47 (8.04)	34 - 85	-	-	-	-
**GeneSTAR (CHARGE)**	CHARGE	Family-based study	European	441	237	50.9 (10.6)	30 - 74	-	-	-	-
**GIG**	ENIGMA	Population-based	European	299	179	24.2 (2.4)	19 - 31	-	-	-	-
**GSP**	ENIGMA	Population-based	European	442	251	21.4 (3.2)	18 - 35	-	-	-	-
**HUBIN**	ENIGMA	Case-control (SCZ and healthy controls)	European	200	70	41.8 (8.1)	19 - 56	104	35	41.6 (8.9)	19 - 56
**IMAGEN**	ENIGMA	Population-based	European	1765	895	14.6 (0.4)	13 - 17	-	-	-	-
**LBC1936**	ENIGMA	Population-based	European	612	289	72.7 (0.7)	71 - 74	-	-	-	-
**LLS**	CHARGE	Family-based study	European	355	187	65.54 (6.65)	55-90	-	-	-	-
**MCIC**	ENIGMA	Case-control (SCZ and healthy controls)	European	170	58	34.0 (11.2)	18 - 60	97	40	32.9 (10.9)	18 - 58
**MooDS**	ENIGMA	Population-based	European	311	164	33.4 (9.8)	18 - 51	-	-	-	-
**MPIP**	ENIGMA	Case-control (MDD and healthy controls)	European	550	318	48.3 (13.3)	18 - 87	177	105	50.1 (12.3)	23 - 78
**NCNG**	ENIGMA	Population-based	European	327	223	51.8 (16.7)	19 - 79	327	223	51.8 (16.7)	19 - 79
**NESDA**	ENIGMA	Case-control (Depression, Anxiety, and healthy controls)	European	231	153	37.8 (10.1)	18 - 57	55	33	41.1 (9.7)	21 - 56
**neuroIMAGE**	ENIGMA	ADHD cases	European	154	23	17.0 (2.5)	11 - 24	Patients-only			
**NTR - Adults**	ENIGMA	Population-	European	400	238	29.7 (10.7)	12 - 56	-	-	-	-

		based Twin Study									
**OATS**	ENIGMA	Population-based Twin study	European	364	238	70.5 (5.1)	65 - 89	-	-	-	-
**PAFIP**	ENIGMA	Case-control (SCZ and healthy controls)	European	117	45	28.4 (8.1)	16 - 51	14	6	24.5 (6.3)	16 - 42
**PROSPER**	CHARGE	RCT/Population-based	European	315	150	74.90 (3.18)	70-82	-	-	-	-
**QTIM**	ENIGMA	Population-based Twin Study	European	845	527	22.5 (3.2)	16 - 30	-	-	-	-
**RSI (CHARGE)**	CHARGE	Population-based	European	939	544	78.9 (4.9)	69 - 96	-	-	-	-
**RSII (CHARGE)**	CHARGE	Population-based	European	1077	569	69.4 (6.0)	60 - 97	-	-	-	-
**RSIII (CHARGE)**	CHARGE	Population-based	European	2397	1333	57.0 (6.3)	45 - 89	-	-	-	-
**RSIx (CHARGE)**	CHARGE	Population-based	European	432	224	72.82 (7.90)	59-90	-	-	-	-
**ROSMAP1**	CHARGE	Population-based	European	184	138	83.19 (6.32)	67-98	-	-	-	-
**ROSMAP2**	CHARGE	Population-based	European	106	83	80.56 (6.88)	60-93	-	-	-	-
**SHIP**	ENIGMA	Population-based	European	966	507	56.4 (12.6)	31 - 90	-	-	-	-
**SHIP-TREND**	ENIGMA	Population-based	European	858	477	50.0 (13.5)	21 - 81	-	-	-	-
**Sydney MAS**	ENIGMA	Population-based	European	543	297	78.4 (4.7)	70 - 90	-	-	-	-
**TOP**	ENIGMA	Case-control (SCZ, BD, other psychoses, and healthy controls)	European	849	407	34.0 (10.4)	17 - 73	305	145	35.4 (9.9)	18 - 73
**UMCU**	ENIGMA	Case-control (SCZ and healthy controls)	European	279	73	31.9 (11.7)	17 - 68	117	44	32.8 (12.8)	17 - 65

**CHAP**	Replication	Population-based	European	261	159	81.02	65-98	-	-	-	-
**LIBD**	Replication	Case-Control (SCZ, healthy control)	European	481	215	33.2 (10.2)	18 - 61	311	175	32.2 (9.9)	18.7 - 61.2
**NIMH-IRP**	Replication	Population-based	European	327	213	34.5 (10.3)	18 - 61	90	66	35.1 (10.3)	19 - 59
**NOMAS**	Replication	Population-based	European	141	69	72.81 (9.11)	50-94	-	-	-	-
**SYS**	Replication	Family-based study	European Founder population	986	510	15.02 (1.84)	11 - 19	-	-	-	-
**TASCOG**	Replication	Replication	Cohort	European	340	142	71.9 (7)	-	-	-	-
**UCLA_NL_BP**	Replication	Case-control (BP and healthy controls)	European	284	150	45.9 (14,3)	19-80	91	49	43.9 (17.1)	19 - 80
**WHICAP**	Replication	Population-based	European	83	51	81 ± 5	70-92	-	-	-	-
**ARIC-Black**	Generalization	Population-based	African-American	389	237	71.6 (4.4)	61-83	-	-	-	-
**CHAP-Black**	Generalization	Population-based	African-American	321	194	78.57	65-97	-	-	-	-
**EDIS-SCES**	Generalization	Population-based	Chinese	210	107	69.88 (6.27)	60-86	-	-	-	-
**EDIS-SiMES**	Generalization	Population-based	Malay	201	106	70.56 (6.65)	60-85	-	-	-	-
**GOBS**	Generalization	Pedigree	Mexican-American	736	456	50.1 (13.3)	26 - 97	-	-	-	-
**NOMAS-Black**	Generalization	Population-based	African-American	168	104	73.55 (8.79)	50-94	-	-	-	-
**NOMAS-Hispanic**	Generalization	Population-based	Mexican-American	718	444	68.97 (8.22)	50-98	-	-	-	-
**Osaka**	Generalization	Case-control (SCZ and healthy controls)	Japanese	545	259	36.1 (12.5)	16 - 71	383	192	36.5 (12.7)	18 - 66
**WHICAP-Black**	Generalization	Case-control (SCZ and healthy controls)	African-American	60	46	81 ± 6	70-97	-	-	-	-

SUPPLEMENTARY NOTE

Case reports

RHEB p.(Pro37Leu) (1) – This male is the first of two children of non-consanguineous parents. His younger sister is similarly affected and is described here as individual 3. Further anamnesis of family history of developmental delay was negative. He was born after 37 weeks of gestation with a birth weight of 3000 gram (0 SD) and APGAR scores of 7 and 8 after 1 and 5 minutes respectively. During his first year, severe hypotonia was present. Neurologic physical examination revealed balance problems, low stretch reflexes and polyneuropathy. An EEG showed diffuse hypofunctional abnormalities, mainly in the posterior brain regions. His motor development was severely delayed. Ultrasound of the cerebrum showed megalencephaly, slightly enlarged ventricles and somewhat cerebellar hypoplasia. He could walk with support at the age of 20 months. Severe pedes plani valgi and a broad based gait were noticed. At the age of 4 years, he could walk unsupported, but this was lost at the age of 5 years due to the development of hip dislocation and kyphoscoliosis. Since that time, the use of a wheelchair is required. There is a lack of speech and there is only minimal non-verbal communication possible. He has a quiet behaviour, but mood changes are present. There are periods of hyperventilation noticed and his sleeping pattern is irregular. Cardiac screening revealed a ventricular tachycardia and ultrasound of the kidneys showed hydronephrosis of the right kidney, requiring surgery. He was diagnosed with gastro-oesophageal reflux, causing oesophagitis (degree II) and requiring medical treatment. Physical examination at the age of 5 years showed macrocephaly with a head circumference of 56.5 cm (+3 SD). Upon evaluation at the age of 29 years, neurologic examination showed spastic tetraplegia, hypotonia and progressive loss of power of the extremities. The patient had severe ID, with absence of speech and used a wheelchair. Physical examination showed a normal head circumference of 60 cm (+1.5 SD) and low height of 171 cm (-2 SD). Telecanthus with a ICD of 4 cm (+3.5 SD) and OCD of 10 cm (+2 SD) were measured. Facial dysmorphisms including frontal bossing, low nasal bridge, prominent nose and broad mouth were present. White, curly hair were observed. Previous genetic testing, comprising karyotyping, analysis of subtelomeric regions, 250k SNP array and Angelman analysis were normal. Using whole exome sequencing, a heterozygous *de novo* mutation in *RHEB* was identified: Chr7 (GRch37); 9.1511880436>A; NM_005614.3:c 110c>T.(p(Pro37Leu)).

RHEB p.(Pro37Leu) (2) – This female is the second of two children of non-consanguineous parents. Her older brother is similarly affected and is described here as individual 2. Further anamnesis of family history of developmental delay was negative. She was born in breech position after 37 weeks of gestation with a birth weight of 2500 gram (-1 SD). Severe hypotonia was present since birth. She had macrocephaly with a head circumference of 50 cm (+3 SD). CT scan of the brain showed megalencephaly and wide ventricles. At the age of 3 years, epilepsy developed. She had tonic-clonic episodes, for which she was treated with Diazepam. Medication was stopped several years later and after that, she developed absence seizures in a frequency of a few times per year. EEG showed diffuse abnormalities with right frontal more irritative focus. Both motor and language development were severely delayed. She could sit without support from the age of 18 months and walk without support from the age of 13 years. She is able to speak a few words. She was diagnosed with a kyphoscoliosis and congenital hipdislocation, the latter requiring surgery. During periods of anxiety or excitement, hyperventilation is present. Upon evaluation at the age of 23 years, regression of motor functions was reported and her behaviour became more aggressive. Physical examination showed short stature with a height of 151 cm (-3 SD) and normal head circumference of 57 cm (+1 SD). Telecanthus/hypertelorism with a ICD of 3.6 cm (+2 SD) and OCD of 2 cm (+2 SD) were measured. Facial dysmorphisms were similar as her older brother, comprising frontal bossing, low nasal bridge, prominent nose and broad mouth. White, curly hair were observed. Previous genetic testing, comprising karyotyping, analysis of subtelomeric regions, 250k SNP array and Angelman analysis were normal. Using whole exome sequencing, a heterozygous *de novo* mutation in *RHEB* was identified: Chr7 (GRch37); 9.1511880436>A; NM_005614.3:c 110c>T.(p(Pro37Leu)).

RHEB p.(Ser68Pro) - This female is the only child of nonconsanguineous parents. There was no family history of developmental delay. She was born after 36+4 weeks of gestation with a birth weight of 3140 gram (1 SD). At 20 weeks of gestation, a large head circumference was detected. At the end of the pregnancy, mother had protein urea. Delivery was in breech position without further complications. At the age of 1 month, her head circumference was 37.2 cm (+1.63 SD). Physical examination at the age of 3.5 years showed a height of 100.5 cm (0 SD) and a head circumference of 53.4 cm (+2.5 SD). She had severe developmental delay from the neonatal age. At the age of 4.5 years, she was not able to walk and could stand for a short while with support. She was using a wheelchair. The patient had lack of speech and showed autistic features: she was fascinated by objects and there was an absence of eye contact. A brain MRI at the age of 1 year and 9 months showed megalencephaly, broad frontal lobes, mild dilatation of lateral ventricles, thick rostrum of the corpus callosum

with relatively smaller splenium small hyperintense region left occipital region, large pons of the brainstem and multiple perivascular Virchow-Robin spaces. Several short periods with loss of consciousness have been reported but the patient has never been formally diagnosed with epilepsy. Upon EEG examination however, frequent abnormalities compatible with epileptiform activity were noted in the right temporal lobe. There were no feeding difficulties, except that fluids were made semi fluid, to prevent choking. She had frequent constipation. The patient was also reported to have facial dysmorphisms, including a large neurocranium, large fontanel, high broad forehead, low set, posteriorly rotated ears, hypotonic face with some drooping of the eyelids, telecanthus with a ICD of 3.6 cm (+3 SD) and OCD of 8.1 cm (+0.25 SD), slightly down slanting of the palpebral fissures, broad nasal bridge, open mouth appearance and a high narrow palate. Neurologic examination revealed axial hypotonia. She had hyperextensible elbows, hips and ankles. There was an obstruction of the right ductus nasolacrimalis and clinodactyly of digitus 5 of her right hand. Previous genetic testing, consisting of 180k array CGH, *FMR1* repeat expansion analysis, *PTEN* and NSD analysis were normal. Using whole exome sequencing, a heterozygous *de novo* mutation in *RHEB* was identified: Chr7(GRCh37):g.151174492A>G; NM_005614.3:c.202T>C (p.(Ser68Pro)).

SUPPLEMENTARY METHODS

Those three paragraphs have been adapted from Adams et al. [1]

ENIGMA and CHARGE

Genetics

Genotyping was performed using different commercial arrays across contributing sites. Both samples and variants underwent stringent quality control procedures based on genetic homogeneity, call rate (< 95%), minor allele frequency (MAF < 0.01), and Hardy-Weinberg Equilibrium (HWE $p<1\times10^{-6}$). Variants passing those thresholds were used as input for imputation to the 1000 Genomes reference panel (phase 1, version 3) using validated software packages (MaCH/minimac, IMPUTE2, BEAGLE, GenABLE). Variants that were poorly imputed (R2 < 0.5) or uncommon (MAF < 0.5%) were removed before meta-analysis. Full details on the site-specific genotyping and quality control can be found in Supplementary Table 2 of Adams et al. [1].

Imaging

Magnetic resonance imaging (MRI) was obtained from different scanners with diverse manufacturers, field strengths, and acquisition protocols. Images were used to estimate milliliters of ICV from automated segmentations. Most sites measured ICV for each participant by multiplying the inverse of the determinant of the transformation matrix required to register the subject's MRI scan to a common template by the template volume (1,948,105 mm3), using the FreeSurfer software. Poorly segmented images were removed after visual inspection. Most sites generated histogram plots to identify any outliers, which were defined as individuals with a volume > 3 SD away from the mean. Statistical outliers were only excluded if the segmentations were deemed improper. More site-specific information related to the imaging is available in Supplementary Table 3 of Adams et al. [1].

GWAS

GWAS of ICV were performed for each site separately, controlling for age, sex, and, when applicable, age2, population stratification variables (MDS / principal components), study site (for multi-site studies only), diagnosis (for case-control studies only). Studies of unrelated individuals performed a linear regression analyses whereas studies of related individuals (ASPSFam, BrainSCALE, ERF, GeneSTAR, GOBS, NeuroIMAGE, NTR-Adults, OATS, QTIM, SYS) used linear mixed models to account for familial relationships. Summary statistics, including effect estimates of the genetic variant with ICV under an additive model, were used to perform a fixed-effects sample size-weighted meta-analysis using METAL[2]. After the final meta-analysis, variants were excluded if only available for fewer than 5,000 individuals. Meta-analyses were stratified by race and done separately for discovery, replication, and generalization samples. Site-specific quantile-quantile plots were generated to inspect presence of genomic inflation.

SUPPLEMENTARY REFERENCES

1 Adams HH, Hibar DP, Chouraki V, Stein JL, Nyquist PA, Renteria ME et al. Novel genetic loci underlying human intracranial volume identified through genome-wide association. Nature neuroscience 2016; 19: 1569-1582.

2 Willer CJ, Li Y & Abecasis GR. METAL: fast and efficient meta-analysis of genomewide association scans. Bioinformatics 2010; 26: 2190-2191.
